# Evaluation of Single Dose and Fractionated Dose of I-131 Radiolabeled Nanoparticles for Triple-Negative Breast Cancer Treatment

**DOI:** 10.3390/biomedicines11082169

**Published:** 2023-08-01

**Authors:** Suphalak Khamruang Marshall, Nutnicha Kaewpradit, Tavadee Mudmarn, Jirassaya Buathong, Palmuk Sriwirote

**Affiliations:** 1 Department of Radiology, Faculty of Medicine, Prince of Songkla University, Songkhla 90110, Thailand; 2 Molecular Imaging and Cyclotron Center, Department of Radiology, Division of Nuclear Medicine, Faculty of Medicine, Prince of Songkla University, Songkhla 90110, Thailand

**Keywords:** biomedicines, breast cancer, cancer therapy, fractionated dose, I-131, nanodelivery, nanoparticle, PLGA, radiation, sodium iodide symporter

## Abstract

Combination chemotherapy is still the standard clinical care for triple-negative breast cancer (TNBC). However, sodium iodide symporter (NIS) uptake by TNBC has opened the potential of NIS as a molecular target for radioiodine theranostic treatments. Radiolabeled poly(lactic-co-glycolic) acid nanocarrier (NINP) was developed for NIS targeted delivery of I-131 to MDA-MB-231 cells to overcome I-131 low uptake in cancer cells and rapid clearance. The NINP diameter of 237 nm has good particle size uniformity and excellent particle stability. Radiochemical purity, radioactive stability, and radiolabeling yield of NINPs over 72 h were >95%. Cytotoxicity confirmed fractionated NINPs over 72 h to be more effective in cell death than single-dose NINP and both single and fractionated Na^131^I. Cellular uptake in a three-dimensional spheroid confirmed that NINP fractionated-dose achieved ~4.8-fold-higher mean fluorescent intensity than Na^131^I and ~2.7-fold greater reduction in cell viability compared to single-dose. The NINP fractionated-dose initiated greater cellular DNA damage to cells than single-dose NINP, resulting in inhibition of cell cycle progression, resulting in cell cycle progression being inhibited by cyclin-dependent kinases, which play a vital role in the control of MDA-MB-231 cell cycle. NINPs are biocompatible with blood, and were found to have no negative impact on red blood cells.

## 1. Introduction

In 2023, the American Cancer Society anticipates that there will be approximately 1.9 million new cancer cases and ~610,000 cancer mortalities in the United States [1], of which 353,510 new cases will be female breast cancer, the most commonly diagnosed cancer in women. Furthermore, the World Health Organization (WHO) forecasts that female deaths from breast cancer worldwide between 2018 and 2040 will increase to 10 million, an increase of 58.3%. Of these, between 10 and 15% of all breast cancers are triple-negative breast cancer (TNBC), with women under the age of 50 and women with a BRCA1 mutation having a higher probability of a TNBC diagnosis. Notably, approximately 90% of cancer patients’ mortalities are caused by metastasis, not the primary tumor [2]. In fact, a meta-analysis by Kuksis et al. revealed that brain metastasis would ultimately develop in approximately one-third of TNBC patients [3]. Furthermore, as lifespan extends and the world population ages, there is an increased risk of secondary thyroid cancer subsequent to breast cancer, and an increased risk of secondary breast cancer subsequent to thyroid cancer. For instance, research by Nielsen et al. established an increased probability of developing breast cancer or thyroid cancer as a secondary malignancy after diagnosis [4].

In nuclear medicine, the therapeutic use of radiopharmaceutical iodine (I-131) is the most widely used radioactive nuclide radionuclide for treating glandular disease such as thyroid, Grave’s disease, solitary hyper-functioning thyroid nodule, toxic multinodular goiter, and differentiated thyroid cancer [5,6]. Furthermore, I-131 is highly radioactive, with a short half-life of 8.0197 days, mostly decaying by beta-emission (606 keV; 90%), has a long particle path length (≤12 mm), and ~1 mm penetration of soft tissue. Additionally, it has a low linear energy transfer (LET) of ~0.25 keV/µm. Therefore, the absorption of the electron radiation energy is close to the radiation source, creating a successful I-131 treatment for cancer. In addition, I-131 emits high-energy gamma radiation (364 keV; 10%), and regularly used in scintigraphy imaging. To target specific cells with radiation, chemical compounds are mixed with I-131 to provide a more personalized treatment [7].

Furthermore, in thyroid neoplasia, the expression of sodium iodide symporter (NIS) and its role is critical for I-131 diagnostics and therapies because it transports iodide in thyrocytes [8]. In thyroid tissue the uptake of radioiodine is the result of the iodine being conveyed by the expressed NIS onto thyroid follicular cell basolateral cellular membranes [9]. Thyroid-stimulating hormones (TSH) are secreted by the pituitary gland and released into the bloodstream, where they regulate thyroid hormone creation such as thyroxine (T4) and triiodothyronine (T3). More specifically, iodine is required for the production of thyroid hormone; its circulating form iodide is absorbed by thyroid follicular cells and oxidized by thyroid peroxidase (TPO) to produce iodine. Consequently, the amalgamation of iodine into the thyroglobulin, called organification, is a biochemical reaction that produces the thyroid hormones, primarily thyroxine (T4) and triiodothyronine (T3) [10]. In addition, the NIS theranostic gene is a promising target for noninvasive radionuclide imaging and therapy. The NIS gene encodes a membrane protein responsible for transporting iodide ions into thyroid follicular cells, where iodide is crucial for thyroid hormone synthesis. However, NIS is not limited to the thyroid and is also expressed in various other tissues, including breast cancer cells. Additionally, NIS has gained significant attention, due to its potential as a theranostic target. The development of NIS-based theranostics holds great promise for improving cancer diagnosis and treatment, offering potential benefits in terms of efficacy, precision, and reduced toxicity [11,12,13]. Moreover, the expression of NIS also occurs in the ciliary body of the eye, choroid plexus, gastric parietal cells, mammary glands, and salivary glands. One of the initial NIS studies looks at prostate cancer prostate-specific antigen (PSA) NIS expression. Expression of NIS resulted in specific I-131 uptake and androgen-sensitive human prostate adenocarcinoma cell (LNCaP) death [14]. As a result, there has been an increased interest in studies applying I-131 for the treatment of other cancers [15,16,17,18]. In particular, there has been growing research into NIS expression as a potential target for I-131 treatment and diagnosis of breast cancer. In particular, a study by Wapnir et al. evaluated the accumulation of NIS-mediated I^−^ in breast cancer metastases, verifying the accumulation of iodide at metastatic sites [19]. Additionally, Chatterjee et al. established that NIS expression was found in 70% of breast cancer cases and that NIS expression differs among the breast cancer subtypes [20]. Furthermore, one of the more recent studies was a HER2-positive breast cancer phase I study to evaluate the biodistribution, radiation dosimetry, tumor imaging ability, and safety of I-131 radionuclide theranostic agents targeting cancers expressing HER2 [21]. Consequently, it determined that the targeted I-131 radionuclide theranostic agent used to treat stage IV HER-2-positive breast cancer patients accumulated specifically in metastatic sites.

Furthermore, TNBC is treated with fractionated radiotherapy, by dividing the radiation dose into smaller fractions and administering it over a number of sessions. Fractionation controls tumors without negative effects, enabling healthy cells to recover between radiation sessions, reducing damage and increasing tissue healing. TNBC fractionated radiation dose depends on the cancer stage, tumor size, location, and patient health. Standard fractional radiation delivers 45–50 Gy in 25–30 fractions over 5–6 weeks. Hypofractionated radiation therapy reduces treatment time by increasing radiation dose to 40–42.5 Gy in 15–16 fractions over 3–4 weeks. Notably, hypofractionation proved as effective as standard fractionation in early-stage breast cancer trials, including TNBC [22].

For many decades, the application of radiopharmaceutical therapy (RPT) has effectively been used for cancer diagnostics and therapy, as it delivers a highly concentrated absorbed dose to the targeted tumor without damaging adjacent healthy tissue [23,24]. Increasingly, research has focused on developing delivery platforms to enhance radionuclide delivery. For instance, nanoparticle (NP) drug delivery systems designed to surmount free therapeutic biological barrier limitations have proven to provide enhanced biodistribution and safety in the delivery of active pharmaceutical ingredients [25]. In particular, RPT is less reliant on identifying agents and signaling pathways than immunotherapy or biological therapy. Notably, 97% of targeted cancer therapy (biological) clinical trials fail [26], as a result of targeting the wrong signaling pathways [27].

Herein, we constructed a NIS-mediated I-131 radiolabeled polylactic-co-glycolic acid (PLGA) nanoparticle (NINP) to evaluate in a three-dimensional model the efficacy of a single-dose versus fractionated I-131 doses. In addition, we aimed to determine the effectiveness of NIS targeted delivery to inhibit MDA-MB-231 breast cancer cells, to overcome I-131 low uptake in cancer cells and rapid clearance.

## 2. Materials and Methods

### 2.1. Materials and Cell Lines

For this research, MDA-MB-231 breast cancer cells were provided by the Biomedical Engineering, Faculty of Medicine, Prince of Songkla University. Na^131^I was acquired from the Institute of Nuclear Technology Thailand. All polymers, buffer solution, bicinchoninic acid (BCA) test kit (Millipore Sigma, St. Louis, MO, USA), MTS Assay Kit (Abcam, Cambridge, MA, USA), LIVE/DEAD^TM^ Cell Imaging Kit (ThermoFisher Scientific, R37601, Waltham, MA, USA), 1,1′-Dioctadecyl-3,3′,3′-Tetramethylindodicarbocyanine (ThermoFisher Scientific, Waltham, MA, USA), fluorescent dye, deionized (DI) water, and solvents, are listed in a previous study [28].

### 2.2. Preparation and Characterization of Na^131^I-loaded Nanoparticle

**Figure 1:**
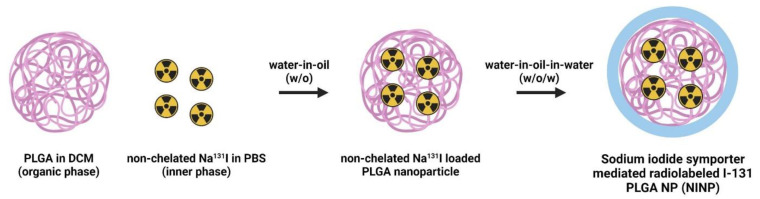
Schematic of NINP nanotherapeutic platform. The fabrication of NIS-mediated I-131 (Na^131^I) radiolabeled poly lactic-co-glycolic acid (PLGA) nanoparticle (NINP).

The protocols for fabricating the poly(d,l-lactide-co-glycolide) nanocarrier (PLGA) polymeric cores by the double emulsion method and radiolabeling the NPs with Na^131^I are provided in a previous study [29]. The double emulsion is commonly used to encapsulate hydrophilic drugs or imaging agents into hydrophobic polymer nanoparticles like PLGA. It involves a series of emulsification steps to form stable water-in-oil-in-water (W/O/W) emulsions (Figure 1).

Step 1: Preparation of the first emulsion (W/O): dissolve PLGA in an organic solvent (dichloromethane, DCM) to form the oil phase, disperse the drug or imaging agent solution in an aqueous phase to form the water phase, slowly add the water phase to the organic phase and sonicate or homogenize to create the first water-in-oil (W/O) emulsion.

Step 2: Formation of the second emulsion (W/O/W): transfer the W/O emulsion obtained in Step 1 into a larger volume of an aqueous phase containing a stabilizer, sonicate or homogenize again to form the water-in-oil-in-water (W/O/W) emulsion, and the W/O/W emulsion stabilizes the encapsulated drug or imaging agent in the PLGA polymer matrix.

Step 3: Nanoparticle formation: evaporate the organic solvent from the W/O/W emulsion to solidify the PLGA nanoparticle cores encapsulating the drug or imaging agent, collect the nanoparticles by centrifugation, and wash them to remove any excess unencapsulated agents.

Furthermore, for radiolabeling the nanoparticles with Na^131^I, the radiolabeling process involves attaching a radioactive isotope, Na^131^I, within the core of the nanoparticles. Na^131^I emits beta particles, which can be used for therapeutic purposes (radiotherapy) or imaging (scintigraphy).

The characterization of the NINP size, polydispersity index (PDI), and zeta potential were attained by dynamic light scattering (DLS) measurements (Malvern ZEN 3600 Zetasizer, EA, The Netherlands) prior to and after loading the PLGA NPs with Na^131^I.

In order to characterize nanoparticle morphology, NINPs were mixed with ethanol to make a homogeneous nanoparticle suspension. The nanoparticle suspension was placed in a small droplet onto the transmission electron microscopy (TEM) grid. The droplet was allowed to air-dry, to ensure uniform distribution of nanoparticles on the grid. Furthermore, staining the nanoparticles using a uranyl acetate contrasting agent enhanced nanoparticle visibility in the TEM images.

### 2.3. Radiochemical Purity of NINPs

Instant thin-layer chromatography (ITLC) (Global Medical Solutions, Bangkok, Thailand) was used to determine Na^131^I standard radiochemical purity percentage (% RCP). A dose calibrator (Capintec, Inc., Ramsey, NJ, USA) was used to calculate the quantity of unbound Na^131^I. Moreover, the contaminants should not account for more than 5% of the total activity. The NINP radiochemical purity was calculated using the following Equation (1):

(1) $$\%RCP=\frac{Total\;counts\;of\;bound\;form\;sample}{Total\;counts\;of\;bound\;form\;sample\;+\;unbound\;form\;of\;NaI}\;\times \;100$$

### 2.4. Radioactive Stability of NINPs

**Figure 2:**
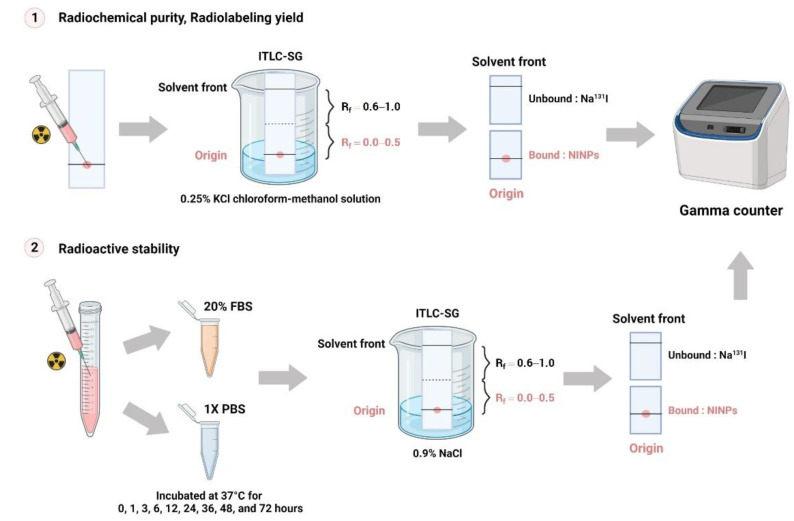
Radiochemical purity, radioactive stability, and radiolabeling yield of NINPs schematic. The standard radiochemical purity percentage (%RCP) of Na^131^I was determined using an instant thin-layer chromatography (ITLC). ITLC chromatography paper strips measured the radiochemical purity, radioactive stability, and radiolabeling yield developing in a 0.25% KCl chloroform-methanol solution. Bound and unbound activity measured by a dose calibrator.

The NINPs were incubated at 37 °C with 20% fetal bovine serum (FBS) and 1 × PBS (Figure 2). Then ITLC-SG was utilized to assess the radiolabeled NPs’ stability, on nine consecutive serum samples with an eluent of 0.9% NaCl solution. To ascertain their stability, the change in the NINP and the radioactive stability was evaluated in triplicate over 72 h.

### 2.5. Radiolabeling Yield of NINPs

The NINP were developed in a 0.25% KCl chloroform-methanol solution over a period of 0, 1, 3, 6, 12, 24, 36, 48, and 72 h, and the Na^131^I radiolabeling yield was quantified by ITLC-SG. Retention factor (R_f_) values were applied to detect radioactive patches. Unbound Na^131^I migrated to the top of the ITLC strip’s top, migrating between R_f_ = 0.6 and 1.0, whereas the bound NINPs migrate at R_f_ = 0.0.

### 2.6. Entrapment Efficacy/Encapsulation Capacity of NINPs

NINPs were synthesized using the double emulsion method described earlier. Evaluating the entrapment efficacy or encapsulation capacity of NINPs involves quantifying the amount of bound and unbound radioactivity and comparing it to the total amount of radioactivity added during NP synthesis. Before beginning the evaluation, perform quality control on the NINPs to ensure they have the desired level of radioactivity. Use the gamma counter to quantify the radioactivity in the nanoparticle suspension. Afterwards, centrifuge the NINPs to separate the NPs from any unencapsulated radioisotope in the supernatant. Furthermore, using a gamma counter, measure the radioactivity associated with NINPs (pellets) obtained after centrifugation. As a result, the total amount of radioactivity associated with the NPs will be provided. Moreover, calculate the encapsulation efficiency (EE%) of the radiolabeled NPs using the following Equation 2

(2) $$Encapsulation\;Efficiency\left(EE\%\right)=\frac{Radioactivity\;of\;encapsulated\;NPs}{Total\;radioactivity\;added}\;\times \;100$$

where radioactivity of encapsulated NPs is the radioactivity measured from the NPs after centrifugation, and total radioactivity added is the initial radioactivity added during NP synthesis.

### 2.7. Radiation Dose Estimation for Individual Wells

Equation (3) was used to determine Na^131^I activity of 3.70 MBq (100 μCi) delivered to each well [29].

(3) $$Ã=\frac{A_{0}\left[\left(1-e^{-\lambda t}\right)\right]}{\lambda }$$

where Ã is the Na^131^I cumulative activity, A_0_ is the initial Na^131^I activity at t = 0, λ is the decay constant, and t is the Na^131^I decay time.

### 2.8. In Vitro Therapeutic Response of NINPs

The MTS assay measures the metabolic activity and viability of cells. When MTS (3-(4,5-dimethylthiazol-2-yl)-5-(3-carboxymethoxyphenyl)-2-(4-sulfophenyl)-2H-tetralium) is mixed with phenazine methosulfate, it changes into a purple formazan. In metabolically active cells, this conversion has been shown to be accomplished by NAD(P)H-dependent dehydrogenase enzymes. The 490 nm absorbance was used to quantify the formazan dye. In addition, the in vitro therapeutic efficacy was ascertained by an MTS. The MDA-MB-231 breast cancer cells were cultivated in Dulbecco’s Modified Eagle Medium (DMEM) (Gibco-BRL, Waltham, MA, USA), augmented with 10% fetal bovine albumin, 1% penicillin/streptomycin (Gibco-BRL, Waltham, MA, USA), and L-glutamine (Gibco-BRL, Waltham, MA, USA). To begin with, MDA-MB-231 cells were cultured to 60–80% confluence at 37 °C, and 5% CO_2_ in culture flasks in a humidified incubator. Afterward, 24 h before treatment, 5000 cells/well were plated onto 96-well microtiter plates at 200 µL/well final volume. Then the MDA-MB-231 cells (*n* = 3) were treated with 1 × PBS, PLGA, single-dose Na^131^I (3.70 MBq), fractionate dose Na^131^I (0.925 MBq × 4), single-dose NINPs (3.70 MBq), and fractionated dose NINPs (0.925 MBq × 4) for 24, 48 and 72 h. Next, the PLGA NP pellets were washed and incubated for 72 h at the final volume of 200 µL/well in the fresh media.

Then, the MDA-MB-231 cell viability was quantified by an MTS viability assay using Equation 4. After this, the well plates were briefly shaken on a shaker, and then a multiplate reader measured both the control cells and the treated cells’ absorbance at OD = 490 nm [30]. Each analysis contains a cell control and a sample control to evaluate cell viability thoroughly. The results were visually illustrated on a graph, by plotting the sample concentration on the x-axis and the cell viability percentage on the y-axis.

(4) $$Cell\;viability(\%)=\frac{ODt-Bg}{ODc-Bg}\times 100$$

where ODt signifies mean optical density of treated cells, ODc the mean optical density of the control cells, and Bg the background mean optical density.

### 2.9. Cellular Uptake of I-131 into In Vitro MDA-MB-231 Cells

The MDA-MB-231 cell surface immunofluorescence and cellular uptake was measured using an ImageStreamX Mk II flow cytometer. The cells were propagated to 80% confluence at 5% CO_2_ in an incubator at 37 °C. The MDA-MB-231 cells were then separated from the culture containers using a solution of Trypsin-EDTA (0.25%). Then 1 × 10^6^ MDA-MB-231 cells were collected by centrifuging them at 4000 rpm for 3 min, and the supernatant was rinsed twice with 1 × PBS at 4 °C. Prior to treatments, DiD red fluorescent dye was encapsulated within the control, PLGA, fractionated-dose Na^131^I (0.925 MBq × 4), and fractionated-dose NINPs (0.925 MBq × 4). Afterwards, MDA-MB-231 single-cell suspensions (*n* = 3) were treated for 1 h with either a control treatment, bare PLGA, fractionated Na^131^I doses, or NINP fractionated-dose. After treatment, cells were washed with 1 × PBS (2 mL), then centrifuged at 350× *g* for 5 min, and washed again. Then, before flow cytometer analysis, the MDA-MB-231 cells were suspended in 1 × PBS. The fluorescence of 5 × 10^4^ cells per sample was measured using a brightfield (gray channel) and DiD (red channel).

### 2.10. Cytotoxicity using LIVE/DEAD^TM^ Cell Method

A two-color fluorescence LIVE/DEAD^TM^ Cell Imaging Kit (488/570) evaluated cytotoxicity and cell viability. The MDA-MB-231 cells were cultured in supplemented DMEM to a 60–80% confluency at 37 °C in a humidified incubator at 5% CO_2_. Prior to treatment, 5000 MDA-MB-231 cells/well were plated, at a final volume of 200 µL/well, into 96-well multiwell plates, for 72 h. The MDA-MB-231 cells (*n* = 3), were then treated in triplicate as follows: control MDA-MB-231 cells (negative control group), PLGA, Na^131^I and NINPs. Previously, the in vitro cytotoxicity study established that 3.70 MBq was the optimum single-dose. Accordingly, the samples were treated with single-dose Na^131^I (3.70 MBq), fractionated-dose Na^131^I (0.925 MBq × 4), single-dose NINPs (3.70 MBq) and fractionated-dose NINPs (0.925 MBq × 4), at 24, 48, and 72 h. After treatment, the culture media was removed and cells washed, then Live Green and Dead Red were mixed, and 50 µL was added to each well plate. To create a 2 × working solution, the Live Green (Comp. A) was mixed with the Dead Red (Comp. B). The equal volumes of 2 × working solution was then added into the MDA-MB-231 cells, control, PLGA, Na^131^I, and NINPs within 2 h, and incubated for 15 min at 25 °C. Afterwards, Trypsin-EDTA (0.25%) solution (Gibco-BRL, Waltham, MA, USA) was added to remove the cells from the culture flasks. Then it was centrifuged to collect the MDA-MB-231 cells, and washed with 1 × PBS, at a temperature of 4 °C. Next, a single-cell suspension in cell staining buffer was prepared and supernatant removed by centrifugation at 350× *g* for 5 min. Afterwards, the treated MDA-MB-231 cells were incubated in the dark at 4 °C for 1 h followed by washing twice with cell staining buffer (2 mL), centrifuged for 5 min at 350× *g*, and then suspended in a buffer and washed twice, prior to flow cytometer analysis.

### 2.11. In Vitro 3D Human Tumor Spheroid LIVE/DEAD^TM^ Cell Imaging

A three-dimensional human MDA-MB-231 tumor spheroid and in vitro LIVE/DEAD^TM^ cell imaging follows the protocol in a previous study [28]. MDA-MB-231 spheroids were treated for 72 h with control, PLGA, single-dose Na^131^I (3.70 MBq), fractionated-dose Na^131^I (0.925 MBq × 4), single-dose NINPs (3.70 MBq) and fractionated-dose NINPs (0.925 MBq × 4).

### 2.12. Level of Apoptosis Incidence and Cell Cycle Analysis of NINPs

To begin with, the MDA-MB-231 cells were seeded at 10^5^–10^6^ cells/well in supplemented DMEM media, and cultured in an incubator with 5% CO_2_ at 37 °C for 24 h. Then the MDA-MB-231 cells were treated for 72 h with control, PLGA, single-dose Na^131^I (3.70 MBq), fractionated-dose Na^131^I (0.925 MBq × 4), single-dose NINPs (3.70 MBq) and fractionated-dose NINPs (0.925 MBq × 4). After the treatment, the supplemented DMEM media were removed, and the cells were washed with 1 × PBS and detached using trypsin/EDTA to remove impurities and form a single-cell suspension.

A fluorometric assay kit (ab39383, Abcam, Cambridge, UK) assessed the caspase-3 activity activated during the apoptosis resulting from the treatments. After collecting the cells, they were re-suspended in a cooled Lysis Buffer I/Cell Lysis Buffer (50 μL), and the cells were then placed on ice for 10 min. After that, to each sample, 2 × Reaction Buffer I/2 (50 μL × Reaction Buffer (10 mM DTT II/DTT) was added. The treated cells were incubated in 5 μL of the 1 mM DEVD-AFC substrate (final concentration 50 μM). Then a fluorescence microtiter plate reader with a 400-nm excitation filter and a 505-nm emission filter measured the cell fluorescence.

Then, cell cycle analysis was assessed; after trypsin dissociation of the MDA-MB-231 cells, they were then rinsed with an assay buffer in order to retain the solution in equilibrium, and the cell pellets were suspended in fresh assay solution (10^6^ cells/mL). Then, prior to propidium iodide staining, the cells were permeabilized with 1 mL of cell fixation agent to terminate further biochemical reactions. Next, the fixation agent was removed by 5 min of centrifugation at 500× *g*. After that, the cells were suspended in a propidium iodide staining solution and then incubated in the dark at room temperature for 30 min. Then cytometer was performed by ImageStreamX Mk II flow to analyze the MDA-MB-231 cell cycle.

### 2.13. Blood Compatibility: Analysis of Hemolytic Properties

The NINP in vitro hemolytic property was assessed with some adjustments, applying Nanotechnology Characterization Laboratory (NCL) protocols [31]. Firstly, human blood was collected with anticoagulant ethylenediaminetetraacetic acid (EDTA) to inhibit clotting, and the plasma was separated and removed from the human blood. Next, 8 × 10^9^ cells/mL human red blood cells were added to the control, PLGA, Na^131^I fractionated-dose (0.925 MBq × 4), and fractionated-dose NINPs (0.925 MBq × 4) for 0, 3, 24, 48, and 72 h, at 37 °C. Afterwards, to ascertain the hemolysis percentage, the blood and NP mixture were centrifuged, and a BCA protein assay kit, at 562 nm, was utilized to quantify the supernatants’ hemoglobin absorbance.

### 2.14. Statistical Analysis

In this study, all of the in vitro experiments were individually replicated three times. The data gathered from the in vitro experiments was stated as a mean ± standard deviation. Additionally, the data normality was evaluated utilizing the residual, and likewise, the variance similarity across the groups was investigated by studying each group variance. Finally, the statistical significance was analyzed using a Student’s *t*-test and a one-way or two-way analysis of variance (ANOVA). The *p*-value < 0.05 were applied to show a statistically significant difference. GraphPad Prism 8.0 software (GraphPad Software Inc., Boston, MA, USA) was used for all statistical analyses.

## 3. Results

### 3.1. NINP Physiochemical Properties and Characterization

**Figure 3:**
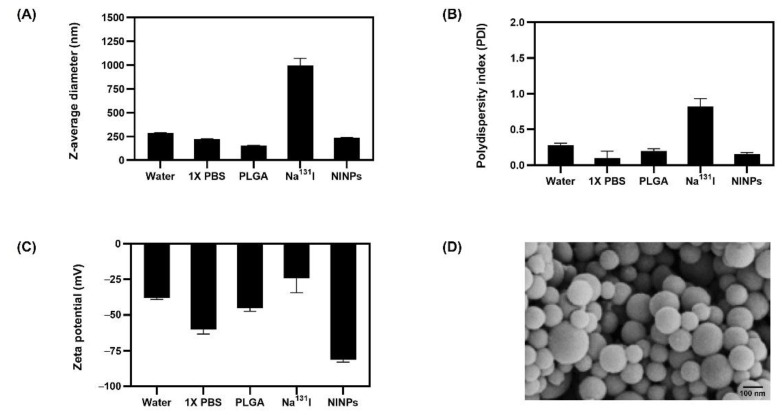
The physiochemical properties and characterization of NINPs. (**A**) Z-average diameter (nm) (**B**) Polydispersity index (PDI) (**C**) Zeta potential (mV) (**D**) Transmission electron micrograph (TEM) image of the NINPs. Scale bar = 100 nm. Data are given as mean ± standard deviation (*n* = 3).

The graph (Figure 3A) shows the z-average diameter (nm), which is the value of the particle size quantified by dynamic light scattering (DLS), indicating a PLGA diameter of 153 nm, and, after Na^131^I radiolabeling, a NINP diameter of 237 nm. The polydispersity index (PDI) graph (Figure 3B) is a measure of the size distribution index. For biomedical applications, a NP PDI below 0.3 is considered acceptable [32]. The results indicated that NINPs have good particle size uniformity, with a PDI of 0.1, suggesting a narrow size distribution, whereas Na^131^I was 0.8 and PLGA 0.2. Additionally, zeta potential, which quantifies the particle’s electrical charge, was measured using DLS, as shown in Figure 3C, which indicated that the stability of the nanoparticles and stability after Na^131^I labeling of the PLGA NPs was good. The results determined the zeta potential values as Na^131^I −24 mV, PLGA −45 mV, and NINP −81 mV, signifying that the NINP has excellent particle stability. In addition, the NINP zeta potential indicates that the particles are highly charged, preventing particle aggregation from electric repulsion [33]. The transmission electron micrograph (TEM) shown in Figure 3D reveals that the NINPs have a hydrophobic poly(lactic-co-glycolic acid) (PLGA) core.

### 3.2. Radiochemical Purity, Radioactive Stability, and Radiolabeling Yield

**Figure 4:**
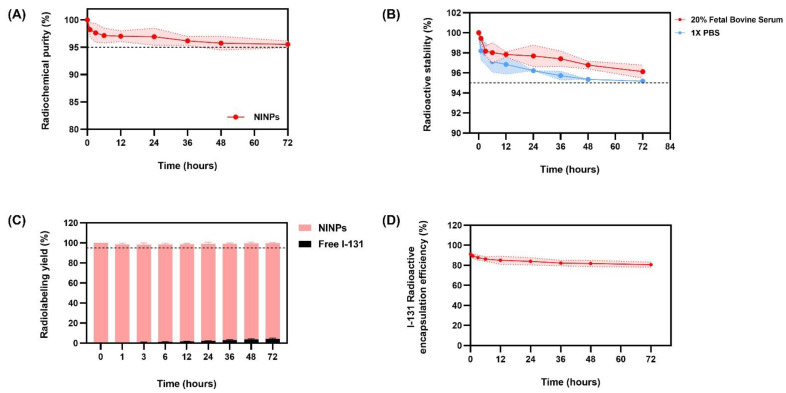
Radiochemical purity, radioactive stability, and radiolabeling yield of the nanoparticles. (**A**) Radiochemical purity (%) of NINPs over 72 h. (**B**) Radioactive stability (%) of NINPs incubated in 20% fetal bovine serum and 1 × PBS over 72 h. (**C**) Radiolabeling yield of NINPs and free I-131 over 72 h. (**D**) Entrapment efficacy/encapsulation capacity of NINPs over 72 h. Radiochemical purity, radioactive stability, and radiolabeling yield should not be less than 95%, according to the World Health Organization Consultation Document guideline. The plotted area is shown as mean ± standard deviation (*n* = 3).

Figure 4A indicates that the radiochemical purity over 72 h was >95%. The radioactive stability over 72 h was >95% (Figure 4B), and the radiolabeling yield at 72 h was >95%, shown in Figure 4C. It is stated that the radiochemical purity, radioactive stability, and radiolabeling yield must be not be less than 95%, according to the World Health Organization Consultation Document guideline [34].

### 3.3. Entrapment Efficacy/Encapsulation Capacity of NINPs

Encapsulation capacity refers to the amount of radiolabel that can be loaded into the nanoparticles per unit weight of the nanoparticles. Therefore, with regard to the NINP encapsulation efficiency, it can be observed that the NINPs have an I-131 encapsulation efficiency of 80–90% over 72 h (Figure 4D).

### 3.4. Optimum Radiation Dose of NINPs In Vitro Therapeutic Response

**Figure 5:**
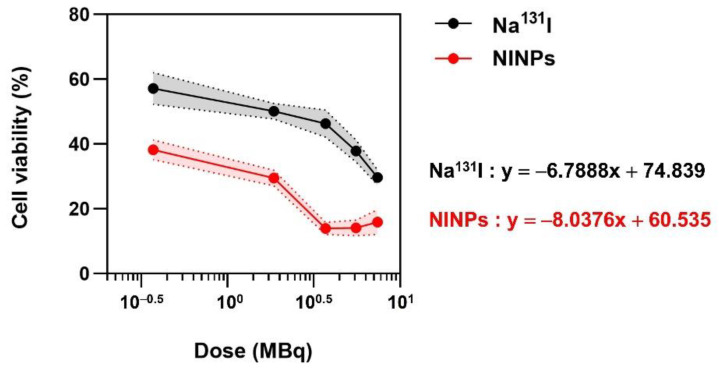
Optimum radiation dose of NINP in vitro therapeutic response. MTS assay in vitro cell viability of Na^131^I and NINPs at dosages of 0, 0.37, 1.85, 3.70, 5.55, and 7.40 MBq, after 24 h incubation. Explanatory variables for the correlation equation y = mx + c: x = radiation treatment dose (MBq) and y = cell viability (%). The plotted area is shown as mean ± standard deviation (*n* = 3).

An MTS assay verified the optimum radiation dose applied in the cytotoxicity evaluation, as illustrated in Figure 5. The graph illustrates the cell viability resulting from different Na^131^I radiation dose levels on MDA-MB-231 cells. The MDA-MB-231 cells were treated with Na^131^I radiolabeled NINPs at incremental radiation doses of 0.37, 1.85, 3.70, 5.55, and 7.40 MBq. It was observed that doses of 3.70, 5.55, and 7.40 MBq resulted in increased MDA-MB-231 cell apoptosis. The MTS assay ascertained that a radiation dose of 3.70 MBq resulted in cell viability of 24%, at 5.55 MBq 24% cell viability, and 27% at 7.40 MBq. It was statistically verified that there was no significant difference in the effectiveness of killing cancer cells (*p* = 0.8895). Accordingly, this study utilized the Na^131^I radiolabeled NINP dose of 3.70 MBq to treat MDA-MB-231 cells.

**Figure 6:**
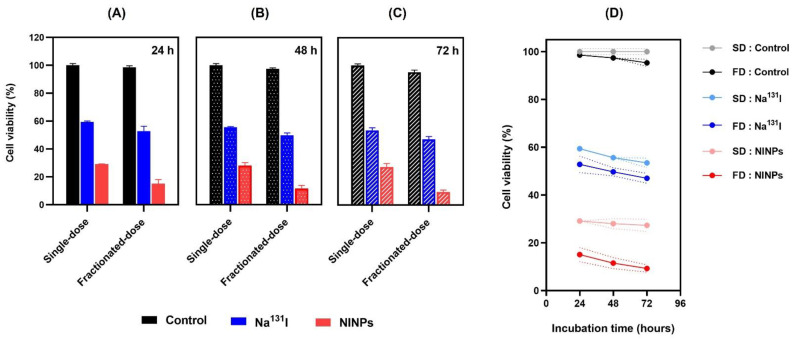
In vitro MDA-MB-231 cell viability after treatment with control, Na^131^I (3.70 MBq), and NINPs (3.70 MBq) at (**A**) 24 h (**B**) 48 h and (**C**) 72 h. (**D**) The comparison of cell viability levels after treatment with control, Na^131^I (3.70 MBq), and NINPs (3.70 MBq), at 24, 48, and 72 h. SD = single-dose (3.70 MBq), FD = fractionated-dose (0.925 MBq × 4). The plotted area is shown as mean ± standard deviation (*n* = 3).

The cell viability graphs (Figure 6) display the results of MDA-MB-231 cell viability after treatment by the control, single-dose Na^131^I (3.70 MBq), fractionated-dose Na^131^I (0.925 MBq × 4), single-dose NINPs (3.70 MBq) and NINPs fractionated-dose (0.925 MBq × 4), at 24, 48, and 72 h. It can be observed that the single-dose and fractionated-dose NINPs had more significant cytotoxicity than the control and Na^131^I, at 24, 48, and 72 h (*p* < 0.0001). At 24 h (Figure 6A), the single-dose Na^131^I cell viability was ~59% and the fractionated-dose Na^131^I ~53% cell viability, in comparison to the single-dose NINPs ~29% cell viability and fractionated-dose NINPs ~15%. At 72 h (Figure 6C), the single-dose Na^131^I cell viability was ~53%, fractionated Na^131^I doses ~47%, the single-dose NINPs ~27% and the fractionated-dose NINPs ~9% cell viability. The results illustrated in Figure 6D verify that at 24, 48, and 72 h, the fractionated-dose NINPs have greater cytotoxicity than the single-dose NINPs and significantly more cytotoxicity than the single and fractionated Na^131^I treatments (*p* < 0.05).

### 3.5. In Vitro Cellular Uptake of I-131 into MDA-MB-231 Cells

**Figure 7:**
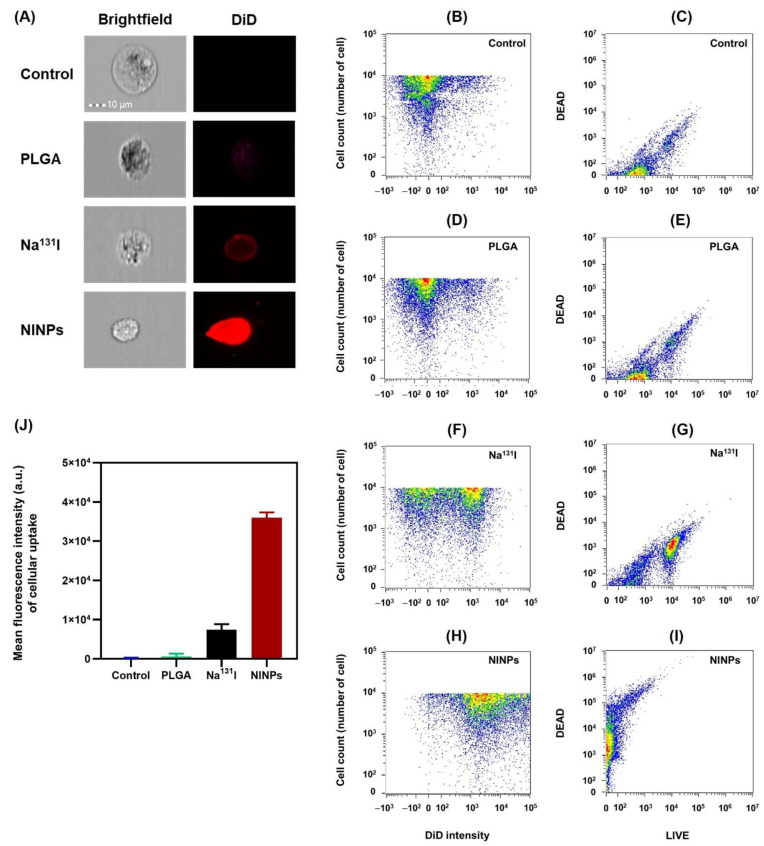
In vitro cellular uptake of I-131 into MDA-MB-231 cells. (**A**) Flow cytometer images of cellular uptake and surface immunofluorescence were collected by brightfield (gray channel) and DiD (red channel) of MDA-MB-231 cells treated with control, PLGA, Na^131^I fractionated-dose (0.925 MBq × 4), and NINP fractionated-dose (0.925 MBq × 4). Scale bar = 10 μm. Scatter plot of DiD cellular uptake and surface immunofluorescence intensity of the MDA-MB-231 cells treated with (**B**) control (**D**) PLGA (**F**) Na^131^I and (**H**) NINPs. Scatter plot of LIVE/DEAD^TM^ immunofluorescence intensity (**C**) control (**E**) PLGA (**G**) Na^131^I and (**I**) NINPs. (**J**) Quantification of DiD (red channel) mean fluorescence intensities of cellular uptake. Data are given as mean ± standard deviation (*n* = 3).

Having established that the in vitro therapeutic response was more efficacious with fractioned doses, brightfield and DiD fluorescence intensity images of MDA-MB-231 cells treated with control, PLGA, fractionated-dose Na^131^I (0.925 MBq × 4), and fractionated NINPs (0.925 MBq × 4) were used to calculate immunofluorescence on the surface of the MDA-MB-231 cells, as illustrated in brightfield (gray channel), and, additionally, the DiD fluorescence (red channel) (Figure 7A). Furthermore, the flow cytometer quantitative investigation of the treatments by the control, Na^131^I, and NINP were implemented to quantify the NINP specific binding to MDA-MB-231 cell receptors. The MDA-MB-231 cells treated by the fractionated Na^131^I discharged a low fluorescent signal, and, in comparison, the MDA-MB-231 cells treated by fractionated-dose NINPs resulted in a significantly stronger fluorescent signal. The images verify the NINP cellular binding/uptake to the MDA-MB-231 cells, indicating that the DiD dyes permeated into the MDA-MB-231 cytosol, suggesting cell penetration is mostly by means of receptor-mediated endocytosis and receptor-mediated membrane fusion. Figure 7 further illustrates that the control and the PLGA NPs resulted in minor fluorescence shifting. In contrast, the NINP treatment resulted in an extensive alteration in the fluorescence shifting (Figure 7E,I). The results (Figure 7J) demonstrate the significantly higher uptake of fractionated-dose NINPs compared to the control and fractionated-dose Na^131^I. The control had the lowest mean fluorescent intensity of 227 a.u., PLGA 5899 a.u., and the NINPs had the highest mean fluorescent intensity of 35,956 a.u. (*p* < 0.05). Moreover, the free Na^131^I fluorescence intensity was 7429 a.u., signifying that the fractionated-dose NINPs have a 4.8-fold-higher mean fluorescent intensity than the fractionated-dose, Na^131^I, with a significant difference of *p* < 0.05.

### 3.6. LIVE/DEAD^TM^ Imaging of MDA-MB-231 Tumor Three-Dimensional Spheroids

**Figure 8:**
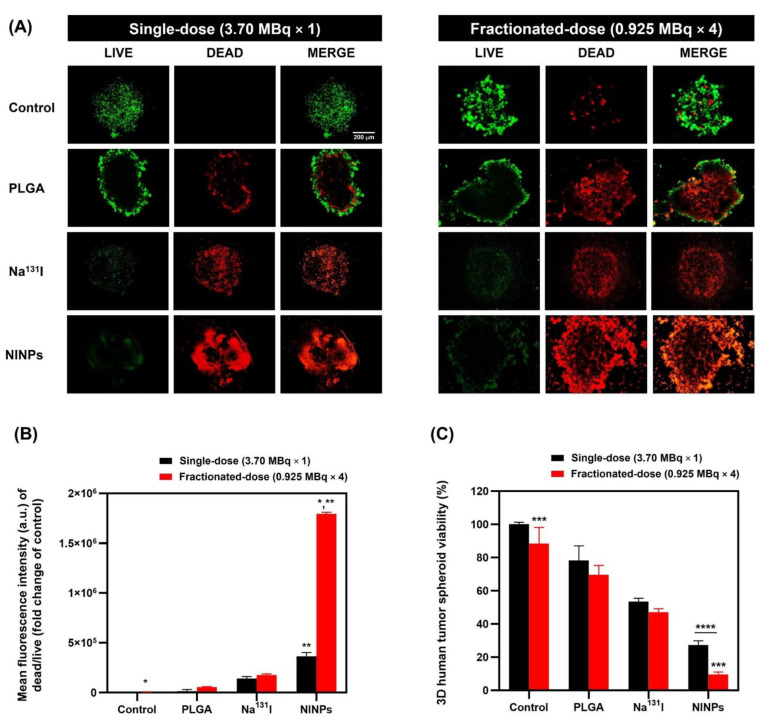
Human tumor spheroids in vitro LIVE/DEAD^TM^ cell imaging in three dimensions (3D). (**A**) LIVE/DEAD^TM^ imaging of MDA-MB-231 tumor spheroids treated with control, PLGA, Na^131^I single-dose (3.70 MBq), Na^131^I fractionated-dose (0.925 MBq × 4), NINPs single-dose (3.70 MBq), and NINP fractionated-dose (0.925 MBq × 4) in vitro for 72 h. LIVE (green channel) and DEAD (red channel) two-color fluorescence allowed for the evaluation of live and dead cells to determine cell viability and cytotoxicity. In addition, all channels were deconvolved by software to eradicate out-of-focus fluorescent signals (10× images, scale bar = 200 μm). (**B**) MDA-MB-231 cell tumor spheroids mean fluorescence intensity of DEAD/LIVE (fold-change of control) after treatment by the control, PLGA, single-dose and 4 × fractionated-dose of Na^131^I, and NINPs for 72 h. * indicates significance in mean fluorescence intensity of dead/live (fold-change of control) between control and NINPs (*p* < 0.05). ** indicates the mean fluorescence intensity of dead/live (fold-change of control) significance between a single-dose and fractionated-dose (*p* < 0.05). (**C**) Three-dimensional MDA-MB-231 human tumor spheroid viability utilizing Cell Titer-Glo^®^ 3D cell assay of cells treated by the control, PLGA, single-dose and 4 × fractionated-dose of Na^131^I, and NINPs for 72 h. *** indicates a significant difference in MDA-MB-231 cell tumor spheroid viability between the control and NINPs (*p* < 0.05). **** indicates a significant difference in MDA-MB-231 cell tumor spheroid viability between single-dose and fractionated-dose treatments (*p* < 0.05). The data are presented as the mean ± standard deviation (*n* = 3).

LIVE/DEAD^TM^ imaging of MDA-MB-231 tumor three-dimensional spheroids treated with single-dose and fractionated-dose is illustrated in Figure 8, indicating the live cells (green channel) fluorescence intensity (a.u.) and dead cells (red channel) fluorescence intensity (a.u.). The mean fluorescent intensity of the NINPs 4 × fractionated-dose was 1,794,257 a.u. and was 4.9-fold-higher than the single-dose NINPs mean fluorescent intensity of 363,916 a.u. Additionally, it was ascertained that the 3D MDA-MB-231 human tumor spheroids treated by the NINP fractionated-dose resulted in a 9.58% cell viability, and in the single-dose NINPs, a cell viability of 27.35% (Figure 8C).

Radionuclides destroy cancer cells preferentially because of their linear energy transfer (LET) and the relative biologic efficacy of the released particles (RBE). The LET property quantifies the amount of ionization when a charged particle traverses a series of cell widths. Na^131^I kills cells principally by beta radiation (606 keV) by triggering apoptosis and necrosis in cancer cells, suppressing cancer development [35]. Additionally, it was shown that Na^131^I treatment might alter the morphologies of cancer cells and boost the efficacy of immunotherapy against cancer cells. In addition, ionized cancer cells exhibit increased expression of the Fas ligand (CD178) and, additionally, the tumor necrosis factor-related apoptotic-inducing ligand (TRAIL) receptor [36].

### 3.7. Level of Apoptosis Incidence and Cell Cycle Analysis of NINPs

**Figure 9:**
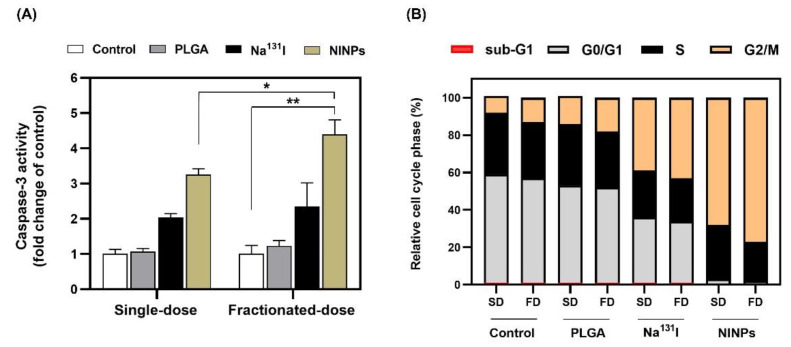
Level of apoptosis incidence and cell cycle analysis. (**A**) Caspase-3 activity (fold change of control) treated with single-dose (3.70 MBq), and fractionated-dose (0.925 MBq × 4) of control, PLGA, Na^131^I, and NINPs in vitro. (**B**) Relative cell cycle phase (%) of MDA-MB-231 in sub-G1, G0/G1, S, and G2/M treated with single-dose, and fractionated-dose of control, PLGA, Na^131^I, and NINPs in vitro. SD = single-dose (3.70 MBq), FD = fractionated-dose (0.925 MBq × 4). * indicates a significant difference of apoptosis incidence level in MDA-MB-231 cell treated with NINPs between single-dose and fractionated-dose (*p* < 0.05). ** indicates a significant difference of apoptosis incidence level in MDA-MB-231 cell treated with fractionated-dose between control and NINPs (*p* < 0.05). The data are presented as the mean ± standard deviation (*n* = 3).

The results of the fluorometric assay to evaluate the caspase-3 activity activated during apoptosis as a result of the various treatments are shown in Figure 9A. The NINPs fractionated-dose caspase-3 activity was 1.14-fold greater than the single-dose NINPs. In addition, the NINPs fractionated-dose were 2.35-fold greater than the single-dose Na^131^I and 2.04-fold greater than the Na^131^I fractionated-dose.

The time-dependent effect of the treated cells’ DNA content was evaluated by a flow cytometer (Figure 9B), illustrating the cell cycle progression after treatment by the control, PLGA, single-dose Na^131^I, Na^131^I 4 × fractionated-dose, single-dose NINPs, and 4 × fractionated-dose NINPs, indicating the MDA-MB-231 cell distribution after each of the various treatments. This revealed that ~58% of MDA-MB-231 cells treated by the control were located in the G0/G1 phase, ~33% of the cells in the S phase and the remaining ~9% in the G2/M phase, whereas treatment with the single Na^131^I dose and the 4 × fractionated-dose Na^131^I resulted in a ~1.70-fold decrease in the percentage of MDA-MB-231 cells in the G0/G1 phase. Moreover, a more significant reduction in the G0/G1 phase can be seen, with 2–3% of cells in the G0/G1 phase after treatment with single-dose NINPs and 4 × fractionated-dose NINPs. Furthermore, the cell distribution in the control increased from 9–13% in the G2/M phase to between 68–77% after single and 4 × fractioned doses NINP treatments. The findings indicate that the NINP treatment increased the proportion of cells in the G2/M phase and additionally decreased the proportion of cells in the G0/G1 phase, compared to the control.

### 3.8. Blood Compatibility: Analysis of Hemolytic Properties

**Figure 10:**
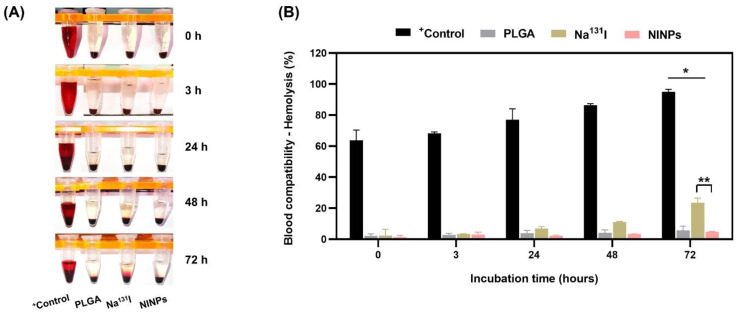
Analysis of nanoparticle in vitro hemolytic properties: 8 × 10^9^ human red blood cells/mL incubated at 37 °C. (**A**) Lysed hemoglobin analysis of red blood cell pellets and supernatant; DMSO (positive control), PLGA, Na^131^I, and NINPs after 0, 3, 24, 48, and 72 h incubation. (**B**) Supernatant hemolysis (%) analysis for lysed hemoglobin; DMSO (positive control), PLGA, Na^131^I, and NINPs after 0, 3, 24, 48, and 72 h incubation. * indicates significant differences in hemolytic characteristics between DMSO (positive control), PLGA, Na^131^I, and NINPs at 72 h (*p* < 0.05). ** indicates significant differences in hemolytic qualities between Na^131^I and NINPs at 72 h (*p* < 0.05). The data are shown as mean ± standard deviation (*n* = 3).

A hemolysis assay was performed to evaluate the treatment’s compatibility with human blood to assess the effect of the positive control, Na^131^I, and NINPs, on human blood, as shown in Figure 10. The positive control hemolysis at 0 h was 64%, increasing to 95% at 72 h. Free Na^131^I, which is frequently used in clinical applications, resulted in hemolysis of 2.33% at 0 h, increasing to 12% at 72 h, proving it produced minimal disruption. In addition, the NINP hemolysis at 0 h was 1%, increasing to 9% at 72 h, indicating that the NINPs caused substantially less damage to red blood cells than both the control and Na^131^I. Furthermore, the hemolysis of Na^131^I in comparison to the NINPs was significantly different (*p* < 0.05). Therefore, the hemolysis assay results signify that the NINP blood biocompatibility does not adversely affect human red blood cells.

## 4. Discussion

The construction of the theranostic sodium iodide symporter-mediated Na^131^I radiolabeled PLGA nanoparticle (NINP) comprises PLGA NP synthesis and radiolabeling with Na^131^I. PLGA degrades to lactic and glycolic acids, providing the necessary biocompatibility and biodegradability for humans; it is licensed for use in drug delivery systems by the US Food and Drug Administration (US FDA), because of its controlled and sustained release qualities, minimal toxicity, and biocompatibility with tissue and cells. The initial z-average diameter of the PLGA NP was 153 nm, increasing to 237 nm after radiolabeling with Na^131^I 997 nm (Figure 3). The significantly larger size Na^131^I can potentially be eliminated from the circulatory system more quickly than the smaller NINPs. In fact, a study by Tian et al. established that after intravenous injection, Na^131^I was rapidly excreted without accumulation in the tumor. However, the smaller diameter I-131 radiolabeled Albumin-Paclitaxel NPs following intravenous injection exhibited prolonged blood circulation time and enhanced tumor penetration and uptake [37]. The NINP zeta potential of −81 mV indicates that the NINP has excellent particle stability. As a zeta potential of 0 to ± 5 results in rapid coagulation or flocculation, ±10 to ±40 is unstable, a zeta potential range of ±40 to ±60 has good stability, and over ±60 has excellent stability [38]. Additionally, Huang et al. investigated NP penetration, accumulation and the influence of NP surface charge by creating a spheroid-on-chip with 3D multicellular spheroids [39]. As a result, they demonstrated that the charge on the surface of NPs influences attachment and penetration, with negatively charged NPs attaching more readily to the spheroid surface and penetrating deeper within the spheroids. In addition, the PDI of 0.1 indicates that the NPs have a narrow size distribution. Furthermore, NP size and surface charge are the most frequently stated factors responsible for cellular uptake [40], and the dissolution of NPs is often critical for deciphering biological responses [41].

Furthermore, radionuclide treatment employs radiopharmaceuticals, which combine a particle-emitting radionuclide with a carrier, targeting the radioactivity to the areas of disease. The primary objective of radionuclide therapy is to maximize cancer cell destruction and spare healthy tissue, to minimize damage to tissue in the surrounding region. There are various methods for radiometal trapping, or radiolabel nanocarriers, including nanocarrier surface labeling, bioconjugation by surface-bound labeling, and aqueous phase trapping during nanoparticle manufacturing. In this research, PLGA NPs were synthesized by the double emulsion method, and at the aqueous phase, radioactive I-131 (Na^131^I) was trapped in the PLGA core. Likewise, nanoliposome internal and external surface radiolabeling was evaluated by Li et al. to treat cervical cancer [42]. They observed that internal radiometal trapped nanocarriers resulted in a greater radiolabeling rate and also in larger radioactivity doses, and achieved superior cytotoxicity than externally radiolabeled nanocarriers. Additionally, the non-chelator procedure incorporating the Na^131^I into the core of the PLGA NP eliminates the requirement for surface chelating agents. Therefore, the non-chelator procedure is more straightforward and quicker than the chelator-based procedure [43].

In addition, Na^131^I ionizing radiation can cause irreversible damage to the nucleus of the MDA-MB-231 cells. In particular, DNA is the principal source of damage produced by ionizing radiation contact. Whether direct or indirect, ionizing radiation can cause molecular damage, including DNA single-strand breaks, double-strand breaks, double-strand break clusters, different forms of DNA base damage, and damage to DNA protein cross-linkages. To emphasize its impact, one DNA double-strand break may be enough to kill a cell if it disables a necessary gene or triggers apoptosis [44]. Moreover, single-strand breakage, cross-links, and base alterations may be readily repaired using the intact strand as a template. However, it is more difficult to repair the damage to clustered lesions, cross-links and double-strand breaks, as it requires additional enzyme repair pathways that are prone to mistakes. As a result of the improper repair, significant implications for the cell and organism might occur. In addition, failed DNA repair contributes to genomic instability, p53-dependent apoptosis, cell cycle halt, and telomere and meiotic abnormalities [45]. Moreover, Na^131^I-ionizing radiation-induced double-strand break clusters are more cytotoxic and mutagenic, and destabilize genomes, as they are extremely difficult to repair when compared to isolated double-strand breaks [46].

Additionally, Na^131^I radiation causes cell death in two distinct forms: apoptosis (programmed cell death) and radiation-induced reproductive failure. First, the ejected electrons in Na^131^I radiation ionize molecules they contact, causing biological damage. As a result, it causes the highly reactive ionized molecules to undergo a surge in chemical changes that cause chemical bonds to break down and disrupt DNA structure. Moreover, ionizing radiation damages all the molecules in the cell by randomly depositing energy. However, most molecules have several copies and recycle rapidly, reducing the damage. Conversely, DNA is a large molecule and a sizeable target for ionizing radiation, with only two copies in each eukaryote cell. Furthermore, the DNA reaction to damage induces complicated DNA damage response pathways that involve cell cycle arrest, which can trigger genes to repair DNA and potentially induce programmed cell death. To emphasize the DNA damage, 1 Gy of irradiation causes about 105 ionizations, damage to 1000 base DNA, and approximately 1000 single-strand and 20–40 double-strand breaks in each cell [47].

Fractionated radiation therapy is a commonly used treatment approach for many types of cancer, including TNBC [48]. It involves delivering radiation in multiple smaller doses over a period of time, instead of a single larger dose. The basic goal of fractionation is to enhance cancer cell death while avoiding collateral harm to healthy tissues. Fractionated radiation therapy has several advantages over single-dose radiation for TNBC cells: (1) Radiosensitivity: TNBC cells tend to be more sensitive to radiation than other breast cancer cells [49]. Fractionated radiation takes advantage of this sensitivity by allowing for the delivery of multiple doses, which can enhance the killing of TNBC cells. (2) DNA repair: fractionation allows healthy cells to repair some of the radiation-induced DNA damage between radiation fractions, while cancer cells, including TNBC cells, may have impaired DNA repair mechanisms [50], resulting in cumulative damage and cell death in cancer cells over the course of treatment. (3) Normal tissue sparing: by delivering smaller doses over several treatment sessions, fractionated radiation therapy helps to spare healthy tissues surrounding the tumor [51]. This is particularly important in breast cancer, as minimizing damage to the heart, lungs, and other critical structures in the chest is a priority. (4) Tumor reoxygenation: fractionated radiation therapy can also promote reoxygenation of the tumor, as the smaller doses allow time for blood vessels to regrow and supply oxygen to the tumor [52]. This is important, because oxygen enhances the effectiveness of radiation in killing cancer cells. Overall, fractionated radiation therapy is more effective than a single high dose in treating TNBC, enabling better tumor control while minimizing side effects.

In this proof-of-concept study, we developed a 3D model to simulate the physical environment of triple-negative breast cancer cells proliferating and interacting with the breast cancer tumor microenvironment (TME) and the extracellular matrix, thereby enabling an improved comprehension of the radiolabeled NINPs’ penetration of the TME and cellular uptake. Three-dimensional cell cultures, particularly multicellular spheroids, have become valuable tools in biomedical research, drug screening, and regenerative medicine. They offer a more realistic and biologically relevant platform for studying various aspects of cell behavior, tissue formation, and disease mechanisms, leading to potentially more accurate and meaningful results than traditional 2D cell cultures. [53]. In addition, 3D multicellular spheroids recapitulate the majority of human solid tumor characteristics in vivo and, consequently, their resistance to treatment [54]. Figure 8B shows the fractionated-dose having a 4.9-fold-higher mean fluorescence intensity than the single-dose. Figure 8C further illustrates that the NINP fractionated-dose treatments resulted in lower cell viability than the NINP single-dose.

Additionally, cell viability (Figure 6) also verified that the NINPs had greater cytotoxicity than the control and Na^131^I, similar to Tian’s findings [37]. In their study, I-131 radiolabeled human serum-paclitaxel NPs, were compared with non-radiolabeled human serum-paclitaxel NPs. They ascertained that the radiolabeled nanoparticles cytotoxicity was greater than that of the non-radiolabeled nanoparticles. In addition, the gamma imaging of nude mice intravenously injected with Na^131^I (7.40 MBq) indicated the signal declined significantly at 5 h, and at 24 h, there was no signal. Moreover, the MDA-MB-231 cellular uptake of the NINPs was demonstrated by the considerably higher DiD fluorescence evenly dispersed throughout the cell cytosol (Figure 7). In contrast, Na^131^I uptake by MDA-MB-231 cells is verified by the red fluorescent dispersal located at the cell’s inner periphery. Suggesting that the NINPs penetrated the MDA-MB-231 cells primarily through receptor-mediated endocytosis and receptor-mediated membrane fusion pathways. Moreover, we verified the significantly higher uptake of the NINPs in comparison to the control and Na^131^I as a result of NIS expression. Additionally, the LIVE/DEAD^TM^ ascertained that fractionated-dose NINPs have ~5.2-fold-higher mean phosphor intensity than Na^131^I, confirming that NINPs are more toxic to MDA-MB-231 cells than Na^131^I (Figure 8).

As shown in Figure 9B, the MDA-MB-231 cell cycle transitions G1/S and G2/M depend on the satisfactory integrity of cells’ DNA. Irradiation of the cells resulted in an increase in cells in the G2/M-phase, signifying the repair of damaged DNA [55], and verifying that the fractionated-dose caused greater DNA damage to cells than the single-dose. This resulted in the cell cycle progression being inhibited by the cyclin-dependent kinases, which play a vital role in the control of the MDA-MB-231 cell cycle. Furthermore, caspase-3 antibodies are extremely useful biomarkers, and are used in this research to monitor the induction of apoptosis to the MDA-MB-231 cells (Figure 9A). Additionally, the caspase-3 enzyme belongs to the family of endoproteases, which are proteins that control the signaling networks involved in apoptosis and inflammation. Because of its involvement in coordinating the destruction of cellular components, such as DNA fragmentation or the degradation of cytoskeletal proteins, caspase-3 is known as an executioner caspase in the process of apoptosis. The caspase-3 activity is carefully controlled, and the enzyme is initially generated in an inactive pro-form as a zymogen [56].

Furthermore, the hemolytic properties of nanoparticles are commonly used in nanoparticle interactions with blood components [57]. In order to determine the degree of induced hemolysis, the hemoglobin concentration was assessed in relation to the total hemoglobin concentration, ascertaining that the NINPs are biocompatible with human blood and were found to have no negative impact on red blood cells (Figure 10).

The next phase of this research is to enhance the targeting ability of this NIS-mediated radiolabeled PLGA nanocarrier, as other organs express NIS and it is imperative to design a nanocarrier with specific targeting, to avoid off-target uptake. There are a number of receptors that are overexpressed by cancer cells, one being the cell surface glycoprotein CD44 that is associated with tumor progression, metastasis, and resistance to therapy [58]. CD44 expression has been found to be higher in breast cancer cells with a more aggressive phenotype, associated with increased invasiveness and resistance to therapy. Therefore, specific targeting of CD44 in breast cancer with monoclonal antibodies, small molecule inhibitors, or siRNA and RNA interference, are possible targeting options [59].

## 5. Conclusions

NIS-mediated I-131 radiolabeled polylactic-co-glycolic acid (PLGA) nanoparticle (NINP) with excellent particle stability was designed with non-chelated radioiodine in the inner core to preserve the nanomaterial integrity, and mediated with a sodium/iodide symporter to assist in radioiodine uptake by MDA-MB-231 triple-negative breast cancer cells. Uptake by the sodium/iodide symporter targeted NINPs and resulted in a ~4.8-fold-greater uptake than Na^131^I. Moreover, the 3D spheroid model revealed that the fractionated-dose NINPs resulted in ~2.7-fold-greater cytotoxicity than the single radiation dose NINPs and ~4.5-fold-greater cytotoxicity than Na^131^I. Additionally, NINP fractionated-dose caspase-3 activity was greater than the single-dose NINPs, single-dose Na^131^I, and fractionated-dose Na^131^I. In addition, cell distribution increased in the G2/M phase after fractioned-dose NINP treatment, and decreased the proportion of cells in the G0/G1 phase compared to the control, PLGA, and Na^131^I. Furthermore, the NINPs are biocompatible with blood, and found to have no negative impact on red blood cells. Additionally, the nanotherapeutic platform successfully delivered fractionated radiotherapeutics, and we believe this nanotheranostic lays the foundation to be developed further for the future treatment and diagnostic of NIS-expressing cancers.

## Data Availability

All data are available upon request to the corresponding author.
